# GENDER DIFFERENCES IN MEANINGFUL LIVED EXPERIENCES RECALLED BY CENTENARIANS

**DOI:** 10.1093/geroni/igz038.1127

**Published:** 2019-11-08

**Authors:** Jyoti Bhatta, Alex J Bishop, Nadia Firdauysa, Tanya Finchum

**Affiliations:** 1 Oklahoma State University, Stillwater, Oklahoma, United States; 2 Oklahoma Oral History Research Program Oklahoma State University, Stillwater, Oklahoma, United States

## Abstract

The purpose of the investigation was to conduct a retrospective examination of meaningful live experiences reported by centenarians. Data for this study originated from N=111 centenarians (n = 43 men; n = 68 women) who participated in the Oklahoma 100-Year Life Project. Applying a hierarchical convoy mapping technique commonly used in social network evaluations, IBM/SPSS 23.0 was used to conduct a descriptive analysis of N = 654 recalled lived experiences. The Mini-Mental Status Examination-SF (MMSE-SF; M = 12.55; SD = 1.55) was used to screen all participants for cognitive orientation prior to participation to ensure capacity to consent and intact memory recall. Centenarian participants recalled a total of M = 6.90; SD= 2.61 lived experiences. Centenarian men recognized a significantly greater average number of meaningful experiences (F = (1, 653) = 30.53, p < .01) compared to centenarian women (M = 4.06 vs. M = 3.43). A good proportion of centenarians (40.50%) acknowledged meaningful events as occurring during young-adulthood. However, the timing of such events occurred significantly earlier (F = (1, 357) = 7.37, p < .01) on average for men compared to women (M = 27.60 yrs. vs. M = 34.11; 1.53). Further analysis revealed that over half of lived experiences considered meaningful among centenarians proportionally fit into three domain types: Family-oriented (19.5%); Work/employment related (18.7%); and Marriage (13.7%). Results have implications relative to understanding how variation in meaningful lived experiences among centenarians. Further evidence of a gendered life course in human longevity will be highlighted.

